# Deep Mycobacterium marinum Infection of the Digit Presenting Without Cutaneous Lesions: A Case Report

**DOI:** 10.7759/cureus.114118

**Published:** 2026-08-07

**Authors:** Wei Jie Tee, Tunku Sara Tunku Ahmad Yahaya

**Affiliations:** 1 Orthopedic Surgery, Changi General Hospital, Singapore, SGP; 2 Orthopedic Surgery, University Malaya Medical Centre, Kuala Lumpur, MYS

**Keywords:** hand infection, hand mass, mycobacterium bovis (m. bovis), non-tuberculous mycobacterial infection hand, tenosynovitis

## Abstract

*Mycobacterium marinum* is a slow-growing nontuberculous mycobacterium acquired through breaches of the skin during contact with fish or aquatic environments. It classically produces a cutaneous papulonodular lesion of the hand, but deeper involvement of tendon sheaths, joints, and bone occurs in a minority of patients and carries greater morbidity and a higher risk of failure of medical therapy alone. We report a rare case in which establishing the diagnosis was challenging: *M. marinum* infection presenting as an isolated deep swelling of a digit without any cutaneous lesion in an immunocompetent fisherman. A 53-year-old man presented with a four-month history of a painful fusiform swelling over the proximal interphalangeal joint (PIPJ) of the right ring finger following a penetrating injury from a fish fin. Plain radiographs showed pressure erosion of the proximal phalanx. Because deep structure involvement is associated with a poorer response to antibiotics alone, the patient underwent excision biopsy and debridement; histopathology demonstrated caseating granulomatous inflammation, and polymerase chain reaction confirmed *M. marinum*. Antimycobacterial therapy with rifampicin and clarithromycin was given for six months. The patient regained PIPJ flexion up to 110° and returned to work pain-free. This case highlights that *M. marinum* should be considered in chronic monoarticular digital swelling with relevant aquatic exposure, even when the characteristic skin lesion is absent, and illustrates the value of tissue biopsy with molecular confirmation and combined surgical and medical management for deep disease.

## Introduction

*Mycobacterium marinum *is a slow-growing nontuberculous mycobacterium that thrives in fresh and marine water and grows optimally between 25°C and 35°C, with survival impaired above 37°C. This may be a thermal preference that explains its predilection for the cooler, distal tissues of the extremities [1,2]. Human infection follows inoculation through a breach in the skin during contact with fish, shellfish, contaminated water, or aquarium maintenance, and the condition is consequently well recognized among fishermen, seafood handlers, and aquarium hobbyists, in whom it is colloquially termed “fish tank granuloma” [1,3]. It is an uncommon infection, and population-based studies report a low incidence [2].

The most frequent presentation is a solitary papulonodular cutaneous lesion of the finger or hand, sometimes with sporotrichoid spread along lymphatic channels [4]. Deeper infection of the tendon sheaths, joints, and bone occurs in a substantial minority of reported cases, at approximately one-third of cases, and is associated with greater morbidity and a higher likelihood of failure of antibiotic therapy without surgery [5-7]. Because the organism grows slowly, is not recovered on routine culture, and is frequently not considered in the differential diagnosis, the interval from symptom onset to diagnosis is often prolonged -- commonly several months [5]. This delay commonly reflects a low index of clinical suspicion, initial misattribution to more common causes of chronic digital swelling such as gout or foreign body reaction, and the frequent failure of standard bacterial cultures to demonstrate growth. Because routine cultures may take weeks to become positive or fail altogether, the laboratory must be alerted to incubate specimens at the lower temperatures the organism favors, and polymerase chain reaction offers a faster and more specific route to species confirmation [4]. Invasive disease is more commonly reported in immunosuppressed patients, such as organ transplant recipients, but it can also affect immunocompetent individuals [8]. We report a case of deep *M. marinum* infection of a digit that is instructive for two reasons: the infection involved deep structures with radiographic bony erosion, and it presented without the characteristic cutaneous lesion, a feature that contributes to diagnostic delay.

## Case presentation

A 53-year-old immunocompetent man, right-hand dominant and a fisherman by occupation, presented with a painful swelling over the proximal interphalangeal joint (PIPJ) of the right ring finger of four months' duration. He recalled a penetrating injury to the digit from a fish fin before the onset of symptoms. The swelling had progressively enlarged and interfered with his daily activities and work. He reported no fever, no constitutional symptoms, and no lymphadenopathy. This was his first presentation for the condition, and he had received no prior treatment. His medical history was unremarkable, with no diabetes mellitus, no immunosuppressive medication, and no history of immunodeficiency. Baseline inflammatory markers, including erythrocyte sedimentation rate and C-reactive protein, were within normal limits.

**Figure 1:**
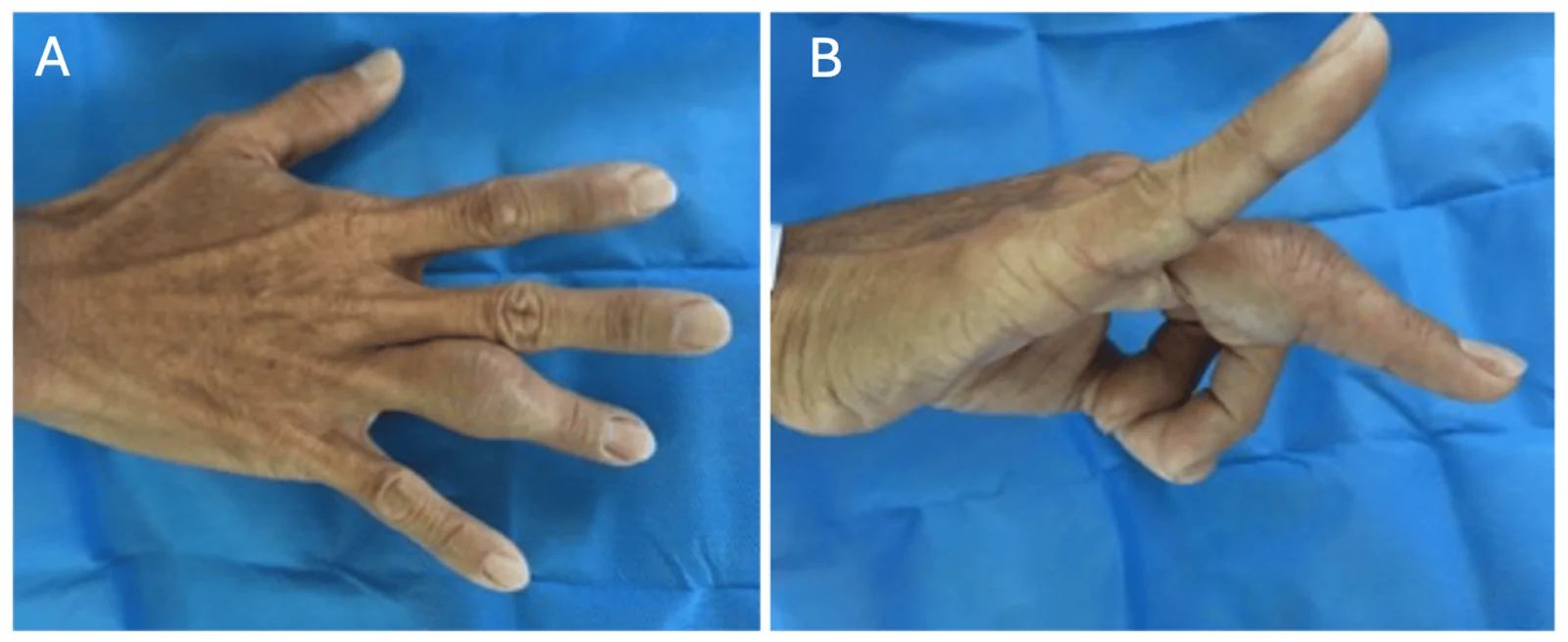
Circumferential fusiform swelling at proximal interphalangeal joint of right ring finger A: Dorsal view of the affected finger B: Ulna view of the right ring finger

On examination, there was a firm, circumferential, fusiform swelling measuring approximately 4 × 3 cm centered on the PIPJ of the right ring finger (Figure 1). There were no overlying skin lesions and no signs of acute inflammation such as erythema, warmth, or discharge. The range of motion at the PIPJ was restricted. The neurovascular examination of the digit was normal.

**Figure 2:**
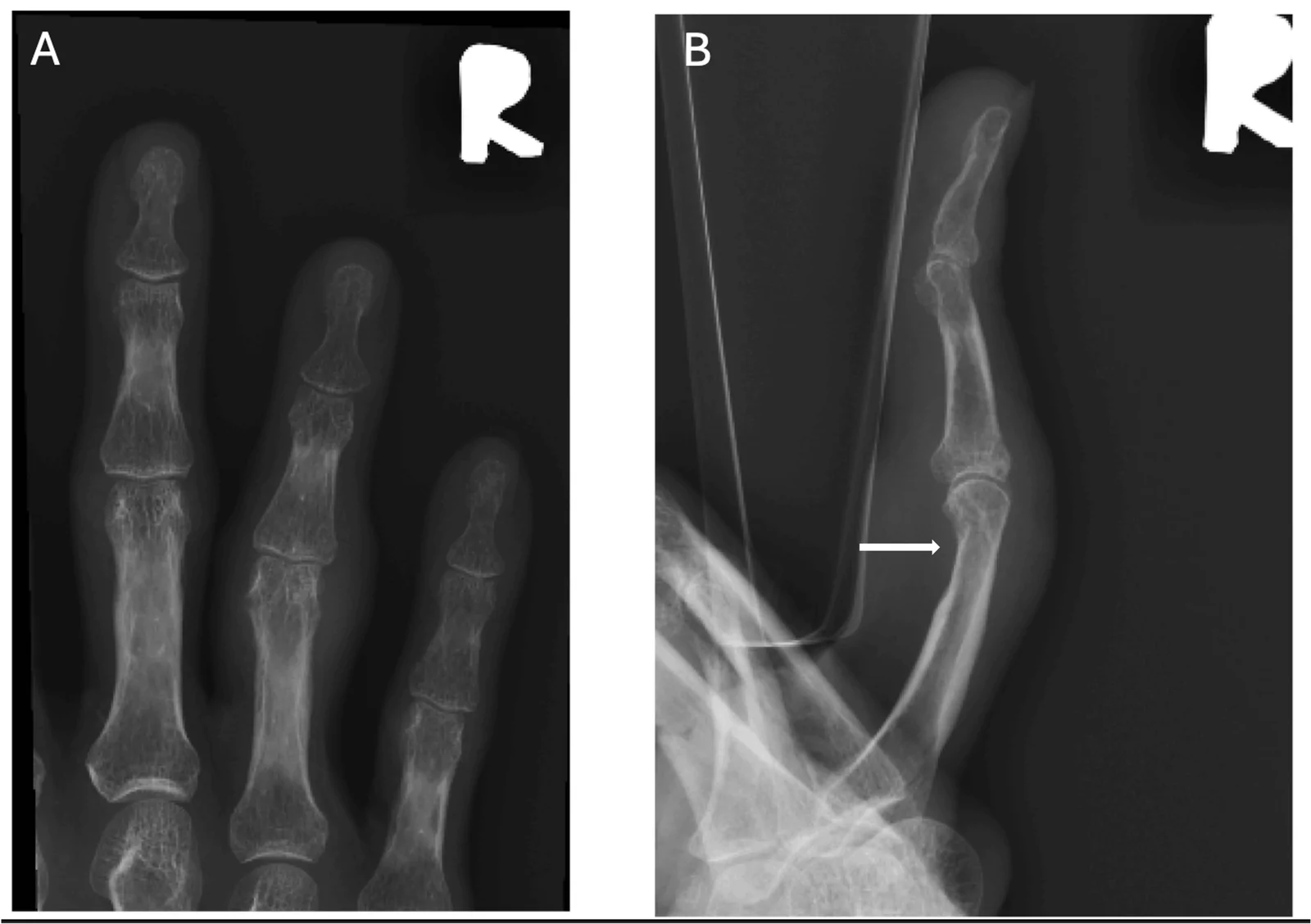
Soft tissue swelling at PIPJ with pressure erosion at head of proximal phalanx of right ring finger A: PA view B: Lateral view demonstrating the area of erosion (white arrow) PA, posteroanterior; PIPJ, proximal interphalangeal joint.

Plain radiographs of the finger demonstrated soft tissue swelling at the PIPJ with pressure erosion of the volar aspect of the head of the proximal phalanx (Figure 2). No magnetic resonance imaging or ultrasonography was performed; imaging assessment was therefore limited to plain radiography.

The differential diagnosis included chronic infective tenosynovitis (mycobacterial, including tuberculous and nontuberculous, and fungal), giant cell tumor of the tendon sheath, gouty tophus, foreign body granuloma, and rheumatoid nodule. In view of deep structure involvement, which is associated with higher rates of failure of antibiotic therapy alone, the patient was counseled for surgery.

**Figure 3:**
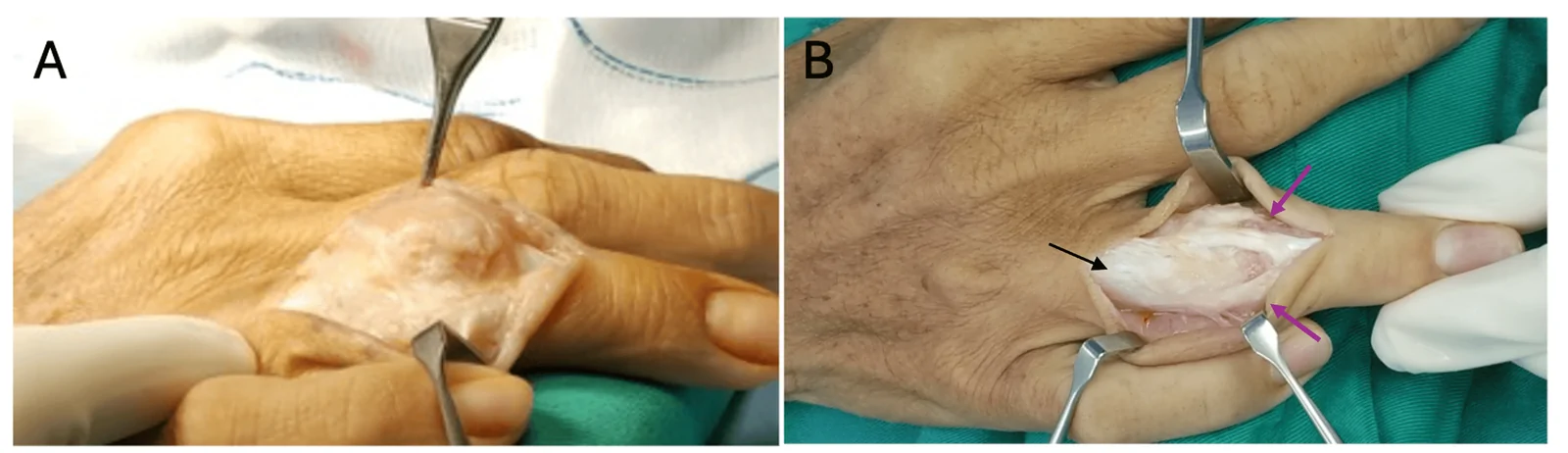
Intraoperative clinical photos A: Inflamed synovial tissues and lesion exposed B: Post-excision photos showing the central slip (black arrow) and lateral bands (purple arrow) were intact

Excision biopsy and debridement were performed. Intraoperatively, inflamed synovial tissue was identified and excised; the central slip and lateral bands were intact (Figure 3). The excised synovial tissue was sent for histopathological examination and for microbiological studies.

**Figure 4:**
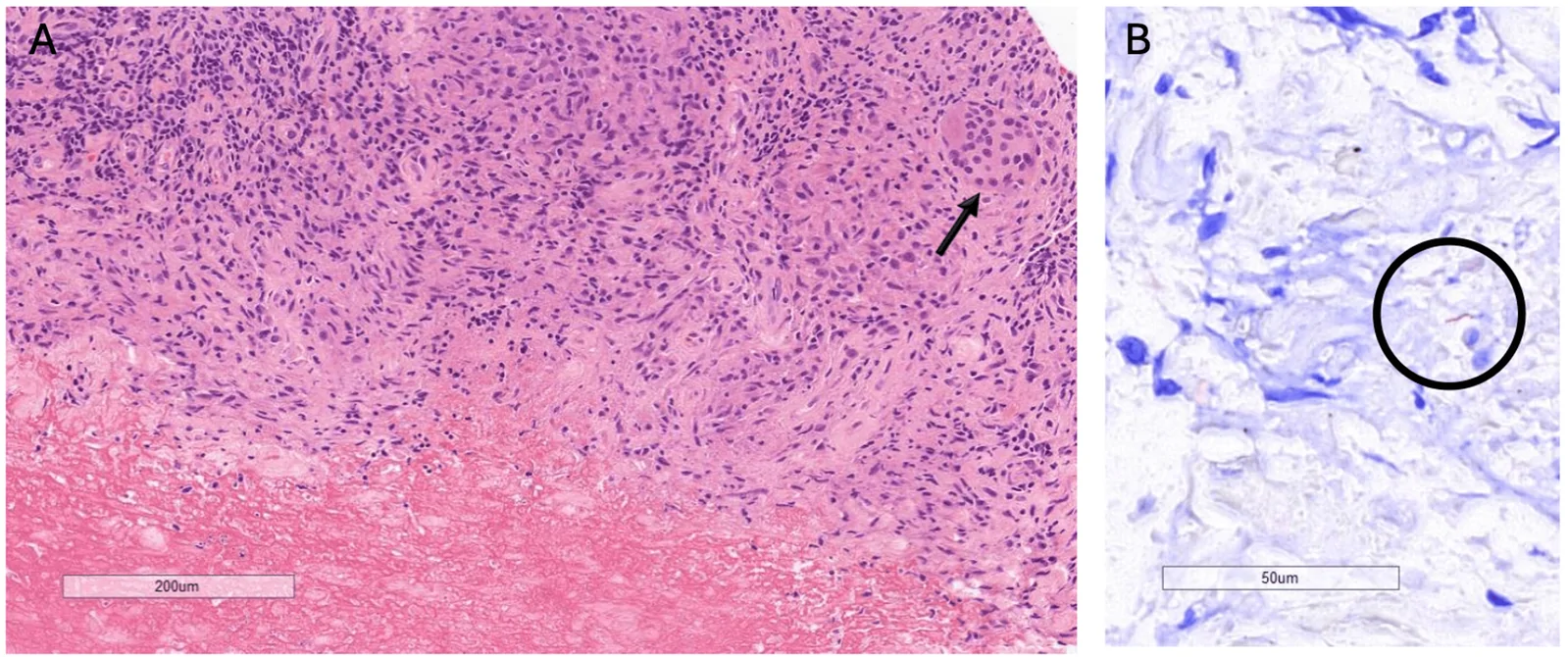
Photomicrographs of intraoperative tissue samples A: Hematoxylin and eosin stain -- necrosis with granuloma and giant cell (arrow) B: Ziehl-Neelsen stain -- acid-fast bacillus

Histopathology showed caseating granulomatous inflammation, consistent with mycobacterial infection (Figure 4). Species identification was established by polymerase chain reaction performed on the excised tissue, supported by the characteristic granulomatous histopathology.

Following confirmation, antimycobacterial therapy was commenced with rifampicin and clarithromycin, in consultation with the infectious diseases team. The wound healed well. At three months after surgery, the patient was able to flex the PIPJ to 110° and had returned to work without pain. He completed six months of antimycobacterial therapy as recommended by the infectious diseases team.

## Discussion

*M. marinum* is among the more common causes of nontuberculous mycobacterial skin and soft-tissue infection, yet remains an uncommon diagnosis overall, and is frequently missed at first presentation [3,5]. Infection of the upper extremity predominates, with the fingers and hand involved in the large majority of reported cases [5]. While most disease is confined to the skin, deeper infection involving the tendon sheaths, joints, and bone is reported in roughly one-fifth to two-fifths of cases and accounts for the greatest morbidity [5]. In retrospective series of invasive disease, tenosynovitis is the dominant deep manifestation, with septic arthritis and osteomyelitis also described, and invasive infection has been more common after fishing- or boating-related exposure than after aquarium exposure [5,6]. Our patient -- an older fisherman with deep PIPJ involvement and radiographic bony erosion -- fits this invasive phenotype.

The most instructive feature of this case is the absence of any cutaneous lesion. The teaching emphasis on the characteristic papulonodular skin lesion can paradoxically delay diagnosis when, as here, the infection presents purely as a deep digital swelling [4]. The diagnostic interval in *M. marinum* infection is characteristically measured in months [5], and our patient's four-month history is consistent with this pattern. A high index of suspicion, anchored on a careful occupational and exposure history, remains the single most important factor in reaching the diagnosis [3].

Diagnosis rests on the combination of a compatible history, histopathology, and microbiological confirmation. Granulomatous inflammation is typical, although the caseation observed in our patient is more characteristic of tuberculosis and can be a source of confusion; molecular confirmation is therefore valuable in distinguishing *M. marinum* from *Mycobacterium tuberculosis* and other nontuberculous mycobacteria. Because *M. marinum* is slow-growing and grows preferentially at lower temperatures, the laboratory must be alerted so that cultures are incubated appropriately; polymerase chain reaction and sequencing allow more rapid and specific identification [4].

There is no standardized regimen for *M. marinum* infection; in their series of 63 cases, Aubry and colleagues noted plainly that “the treatment is not standardized” and found that the choice of antibiotic, surgery, or other modality did not significantly affect the outcome [3]. Most recommend combination antimicrobial therapy as first-line treatment, commonly incorporating a macrolide such as clarithromycin, with surgical debridement reserved for deep infection or for disease that fails to respond to medical therapy [3]. In the present case, deep structure involvement with bony erosion prompted early surgical debridement in addition to antimicrobial therapy, with a good functional result. The duration of therapy is individualized; treatment is generally continued for several weeks beyond clinical resolution, and total courses of several months are usual in deep disease.

The limitations of this report include the absence of advanced cross-sectional imaging, as neither magnetic resonance imaging nor ultrasonography was performed, and the absence of mycobacterial culture and antimicrobial susceptibility data, such that species identification rested on histopathology and polymerase chain reaction. The latter is a recognized and acceptable diagnostic route, given that culture is positive in only 40% to 60% of cases [2]. Nonetheless, this report reinforces a clinically important point: deep *M. marinum* infection can occur without the cutaneous findings most clinicians associate with the disease, and prompt tissue biopsy with molecular testing, combined with surgical debridement where deep structures are involved, enables accurate diagnosis and a favorable outcome.

## Conclusions

A high index of suspicion is essential for the timely diagnosis of *M. marinum* infection of the hand, particularly because the disease can involve deep structures and may present without the cutaneous lesion that classically characterizes it. A careful occupational and exposure history, together with tissue biopsy for histopathology and molecular identification, is key to establishing the diagnosis in chronic digital swelling. When deep structures are involved, combined surgical debridement and antimicrobial therapy can achieve resolution of infection with restoration of function, as in our patient. Individuals at occupational or recreational risk should be made aware of the condition and counseled on preventive measures such as wearing protective gloves when handling fish and aquatic materials.
